# How to rectify the convex coronal imbalance in patients with unstable dystrophic scoliosis secondary to type I neurofibromatosis: experience from a case series

**DOI:** 10.1186/s12891-022-05321-w

**Published:** 2022-04-20

**Authors:** Saihu Mao, Song Li, Yanyu Ma, Ben-long Shi, Zhen Liu, Ze-zhang Zhu, Jun Qiao, Yong Qiu

**Affiliations:** Division of Spine Surgery, Department of Orthopedic Surgery, Nanjing Drum Tower Hospital, Nanjing University Medical School, Zhongshan Road 321, Nanjing, 210008 China

**Keywords:** Convex coronal imbalance, Dystrophic scoliosis, Type I Neurofibromatosis, Upper instrumented vertebra

## Abstract

**Background:**

There was a paucity of valid information on how to rectify the convex coronal imbalance effectively in dystrophic scoliosis secondary to Type I neurofibromatosis (DS-NF1), while postoperative inadvertent aggravation of CCI occurred regularly resulting in poor patient satisfaction. We aimed to identify the risk factors for persistent postoperative CCI in DS-NF1, and to optimize the coronal rebalancing strategies based on the lessons learned from this rare case series.

**Methods:**

NF1-related scoliosis database was reviewed and those with significant CCI (> 3 cm) were identified, sorted and the outcomes of surgical coronal rebalance were analyzed to identify the factors being responsible for failure of CCI correction.

**Results:**

CCI with dystrophic thoracolumbar/lumbar apex was prone to remain uncorrected (7 failure cases in 11) when compared to those with thoracic apex (0 failure cases in 4) (63.6% vs. 0.0%, *p* = 0.077). Further comparison between those with and without post-op CCI showed a higher correction of main curve Cobb angle (65.9 ± 9.1% vs. 51.5 ± 37.3%, *p* = 0.040), more tilted instrumentation (10.3 ± 3.6° vs. 3.2 ± 3.1°, *p* = 0.001) and reverse tilt and translation of upper instrumented vertebra (UIV) to convex side (8.0 ± 2.3° vs. -3.4 ± 5.9°, *p* < 0.001; 35.4 ± 6.9 mm vs. 12.3 ± 13.1 mm, *p* = 0.001) in the uncorrected imbalanced group. Multiple linear regression analysis revealed that △UIV translation (pre- to post-operation) (β = 0.832; *p* = 0.030) was significantly correlated with the correction of CBD.

**Conclusion:**

Thoracolumbar/lumbar CCI in dystrophic scoliosis was prone to suffer high risk of persistent post-op CCI. Satisfying coronal rebalance should avoid UIV tilt and translation to the convex side, tilted morphology of instrumentation and over correction maneuvers for main curve, the upper hemi-curve region in particular.

## Introduction

Dystrophic scoliosis secondary to neurofibromatosis type I (DS-NF1) is characterized by distinctive bone abnormalities causing spinal instability, deformity onset and worsening [1, 2]. Extraordinary and rapid curve progression is common for juvenile patients or those exhibiting ≥3 dystrophic features [3]. A highly rotated short curve span often co-exists with regional kyphosis, while the coronal and sagittal malalignments are prevailing in presence of dystrophic vertebral rotatory subluxation. Trunk shift with subsequent coronal imbalance (CI) can occur in those with highly dystrophic kyphoscoliosis, especially when the curve apex locates in the thoracolumbar/lumbar regions. This represents an additional level of complexity when surgery is indicated to restore the spinal alignments.

Residual or even aggravated CI after surgery has been reported to compromise the health-related quality of life (HRQoL) and can increase the risk of implant failure [4–6]. However, how to effectively rectify the coronal malalignment remains subject of debate. In 2016, Qiu proposed a novel classification for CI in degenerative lumbar scoliosis [7], which indicated that type C CI with coronal balance distance ≥3 cm harbored high risk of immediate post-operative CI. Obeid emphasized that the rebalance of coronal trunk for convex CI depended mainly on the correction of the lumbosacral fractional curve rather than the main curve [8]. Additionally, multiple innovative techniques have been also developed for better restoration of coronal malalignment involving the sequential correction technique, the kickstand and tie rod techniques [9–11]. All these techniques utilize the robust pelvic fixation to provide additional corrective force to obtain marked coronal rebalance.

Despite being effective, spinal-pelvic fixation is only indicated for a limited spectrum of pediatric spinal disorders mainly involving lumbosacral congenital vertebral malformations and neuromuscular scoliosis with pelvic obliquity [12, 13]. For young NF-1 dystrophic scoliosis patients whose lumbosacral discs and facet joints are not degenerated and coronally mobile, pelvic fixation is not the mainstay treatment option [14]. This will be beneficial for preserving the mobility of lumbosacral and sacroiliac joints, retaining the distal coronal compensatory capability and improving the HRQoL [8]. Thus, these aforementioned coronal rebalancing strategies, which are mainly designed for degenerative lumbar scoliosis, can’t be applied indiscriminately to young patients with CI. This dilemma makes the complete depiction of tips for effective correction of convex CI (CCI) in dystrophic scoliosis essential for improving the outcome of spinal realignment.

From the existing literature, to the best of our knowledge, there was a paucity of valid information guiding how to intervene effectively with CCI in DS-NF1. This study was designed to identify the risk factors for persistent postoperative CCI and to optimize the coronal rebalancing maneuvers based on the lessons learned from this case series. We also tried to make a preliminary stratification of convex coronal imbalance to improve the understanding of failed reconstruction of CCI.

## Materials and methods

Following the Hospital Clinical Research Ethics Committee approval, this retrospective study was conducted on patients with DS-NF1 referred for corrective surgery at our institution from October 2011 to November 2018. The diagnosis of dystrophic scoliosis in NF-1 was made using established diagnostic criteria [15, 16]. Enrollment was limited to NF-1 patients with (1) dystrophic scoliosis and concomitant trunk shift causing convex coronal imbalance: coronal balance distance ≥3 cm; (2) intact neurological function before surgery; (3) minimum two-year follow-up with complete image data. The exclusion criteria were applied to those with (1) multiple sporadic dystrophic bone defects along the spine causing double or triple curves; (2) solitary dystrophic lesion in sacrum causing compensatory lumbar/thoracolumbar scoliosis and trunk shift; (3) presence of pelvic obliquity due to dystrophic bone defects in the lower limbs. MRI for each patient was routinely implemented for evaluating the spinal cord and the NF1 associated tumor. A total 179 DS patients with NF-1 were operated during that time period, and finally, only 15 patients (age, 14.7 ± 4.4 yrs.; range, 10-26 yrs.; 7 males and 8 females; mean follow-up, 3.3 ± 1.5 yrs) who fulfilled the inclusion and exclusion criteria were enrolled in this study. Their medical records, imaging scans, and operative reports were reviewed. The data collected include preoperative, postoperative and final main curve Cobb and kyphotic angles, patterns of convex CI, apex location, presence of vertebral rotatory subluxation [17], coronal balance distance (CBD), sagittal vertical axis (SVA), surgical strategies, fusion segments, implant density, ratio of laminar hook, postoperative neurological status, and surgical complications. Curve flexibility was not assessed for this special patient subgroup. This was attributable to the potential risk of neurological impairments if side bending movements were performed hinging on the unstable apical region.

**Fig. 1:**
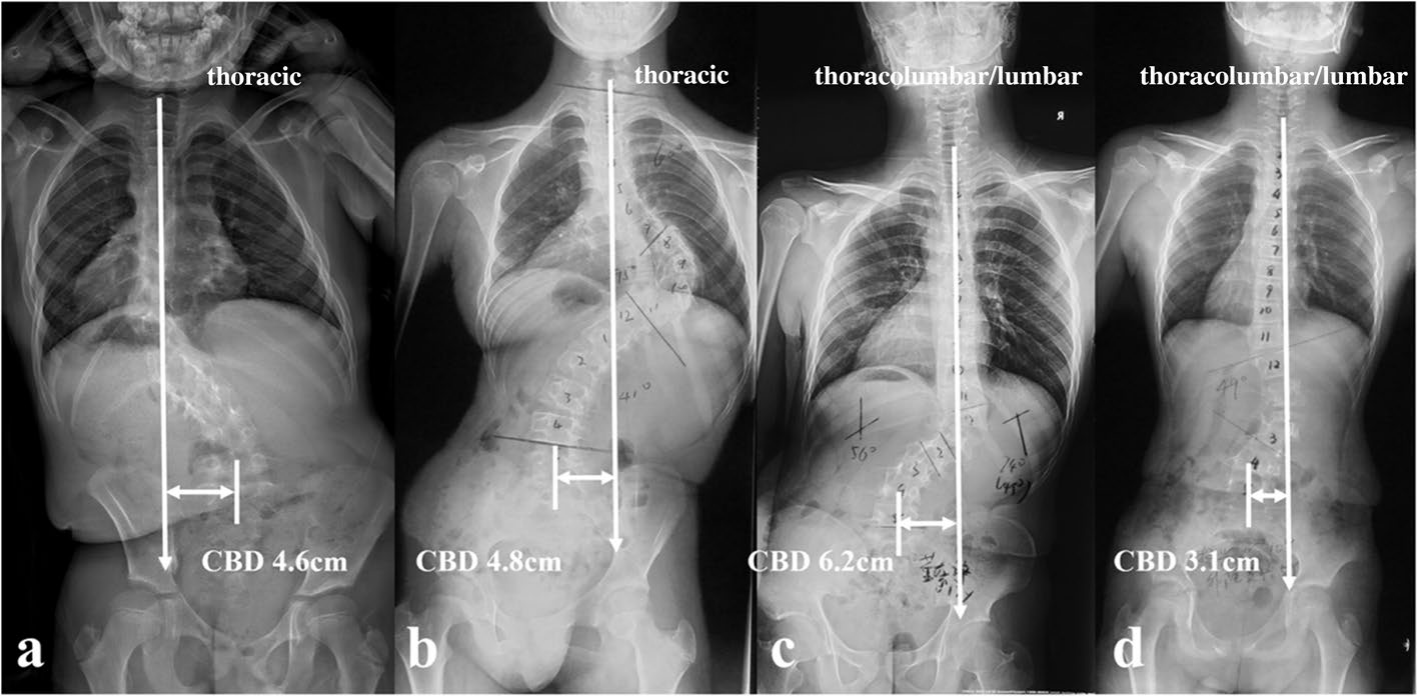
Illustration of the stratification of CCI in DS-NF1: **a** Thoracic apex with vertical proximal hemi-curve; **b** Thoracic apex with tilted proximal hemi-curve; **c** Thoracolumbar/lumbar apex with vertical proximal hemi-curve; **d** Thoracolumbar/lumbar apex with tilted proximal hemi-curve

**Fig. 2:**
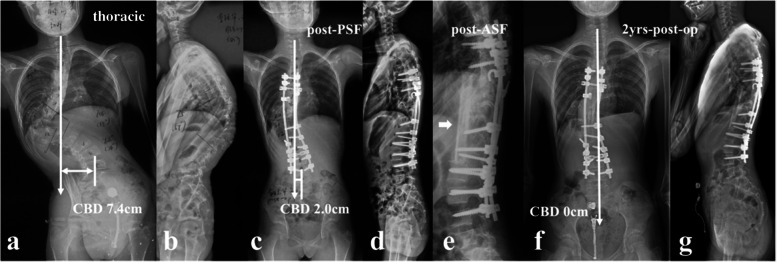
an 11-year-old girl with NF1, suffering from kyphoscoliosis associated with convex trunk shift and dystrophic vertebral rotatory subluxation at T9/10 level (thoracic type) (**a**, **b**); Halo Gravity Traction on wheelchair was prescribed for 1 month. Afterwards, stage 1 posterior spinal correction and fusion surgery was performed (**c**, **d**), followed by stage 2 supplementary anterior fusion utilizing structural fibular allograft (**e**, arrow). 2-year-follow up revealed spontaneous improvement of coronal balance with solid fusion (**f**, **g**)

**Fig. 3:**
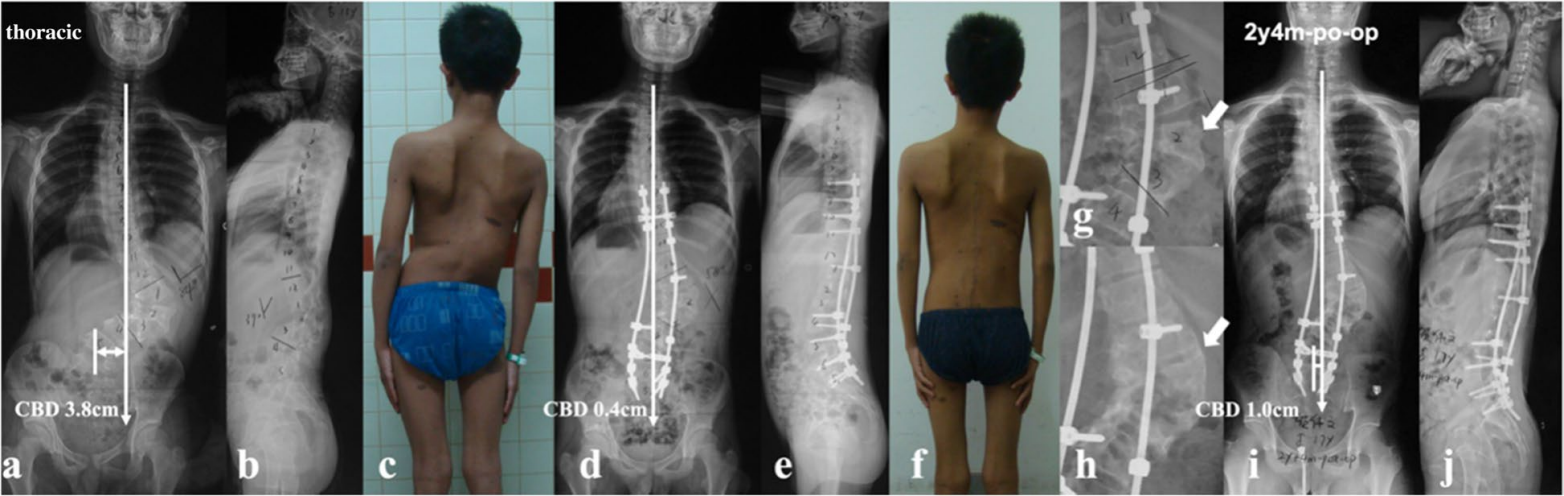
a 14-year-old boy with NF1 related lumbar kyphoscoliosis associated with CCI and dystrophic vertebral rotatory subluxation at L2/3 & L3/4 levels (lumbar type) (**a**, **b** and **c**). Stage 1 posterior spinal correction and fusion surgery was performed with low screw density yet with generous posterior fusion, followed by stage 2 supplementary anterior intervertebral fusion utilizing autogenous rib grafts (**d**, **e**, **g**). The coronal balance was well reconstructed despite low correction rate of main curve (**d**, **f**). 2.25-year-follow up revealed satisfying intervertebral fusion (**h**) and well maintenance of coronal balance with no instrumentation failure (**i**, **j**)

All the recruited patients were stratified according to the location of dystrophic curve apex: thoracic group, 4 cases (26.7%) (Fig. 1a, b); thoracolumbar/lumbar group, 11 cases (73.3%) (Fig. 1c, d). Among them, 1 patient in thoracic group and 3 patients in thoracolumbar/lumbar group received staged surgery with combined posterior-anterior or anterior- posterior approach (Table 1), while the rest 11 patients (73.3%) underwent posterior-only spinal instrumentation and fusion. Supplementary anterior fusion utilizing structural fibular allograft (2 patients) (Figs. 2, 8) or autogenous rib grafts (1 patient) (Fig. 3) was applied when the pedicle screw density in the apical region was distinctively low due to pedicle dystrophy disabling screw insertion. Stage 1 anterior release involving intervertebral disc resection and autogenous rib grafting was performed in 1 patient of thoracolumbar/lumbar group, followed by skull-femoral traction for 2 weeks and subsequent stage 2 posterior spinal correction and fusion.

**Table 1:** Summary of surgical strategies

Surgical strategies	Thoracic (4)	Thoracolumbar/lumbar (11)
PSF	1	8
Skull-Femoral Traction + PSF	1	0
Halo Gravity Traction + PSF	1	0
PSF + ASF	0	2
Halo Gravity Traction + PSF + ASF	1	0
Anterior release + Skull-Femoral Traction + PSF	0	1
Ratio of traction (%)	75%	9.1%
Ratio of combined approach (%)	25%	27.3%

*PSF* posterior spinal fusion, *ASF* anterior supplemental fusion

Spinal traction was indicated if Cobb angle > 90° or kyphosis > 80°. Aside from the aforementioned one patient in thoracolumbar/lumbar group (9.1%), spinal traction was also applied for another 3 patients in thoracic group (75%). Among them, two received halo-gravity traction using a halo-wheelchair for 1 month, while the third one was applied with skull-femoral traction in bed for 2 weeks before posterior surgery. Posterior-only spinal instrumentation and fusion was performed with all pedicle screw or hybrid constructs, and was assisted with satellite rod technique for two patients being operated in late stage.

### Radiographic assessments

**Fig. 4:**
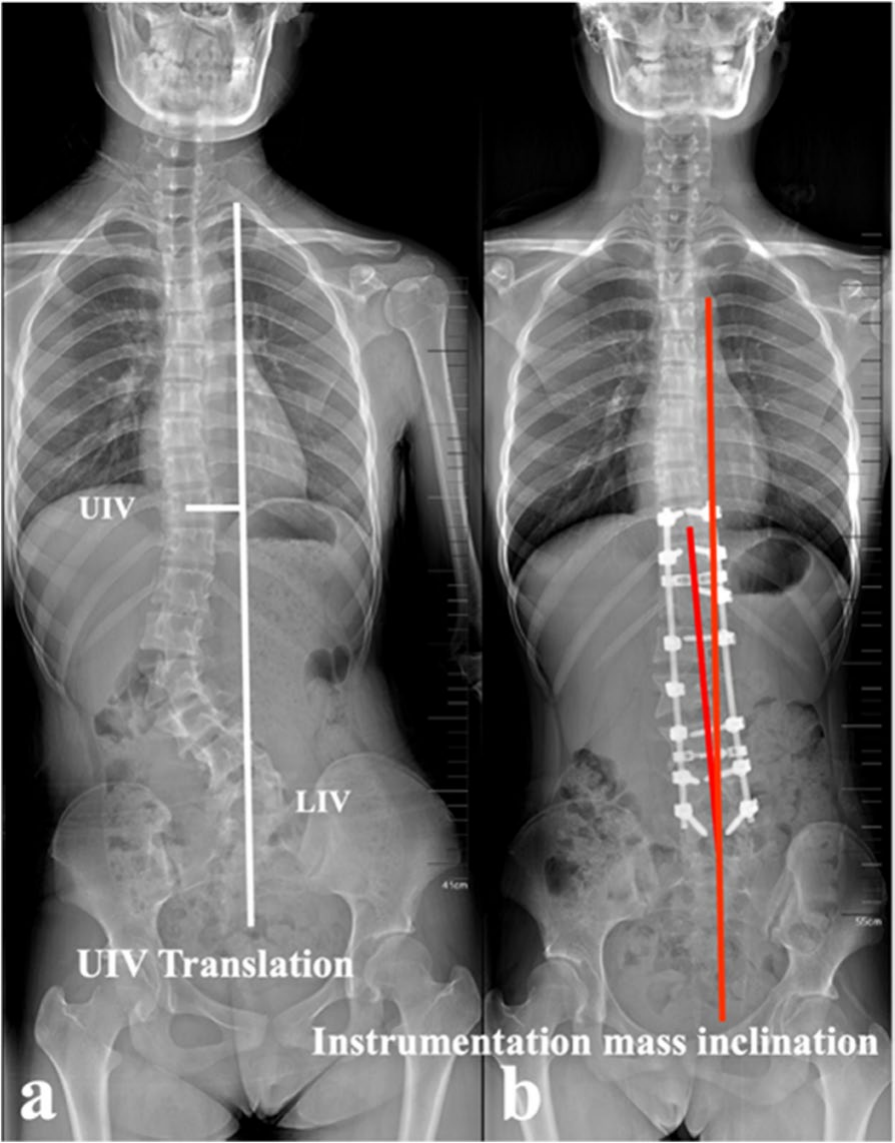
Illustration of the measurements of UIV translation (**a**) and instrumentation mass inclination (**b**)

Radiographic parameters were measured pre- and post-operatively for analysis of CCI prognosis, involving main curve Cobb angle, CBD, UIV (upper instrumented vertebra) tilt, LIV (lower instrumented vertebra) tilt, UIV translation and instrumentation mass inclination. All measurements were performed using the Surgimap spine software (Version 2.3.1.5; Spine Software, New York, NY). The definitions of the standard measuring techniques of the aforementioned parameters were defined as follows:

1. UIV tilt: defined as the angle formed by the line drawn parallel to the superior end plate of the UIV and the horizontal line. A positive value was defined as the UIV inclining to the convexity of the main curve.
2. LIV tilt: defined as the angle formed by the line drawn parallel to the superior end plate of the LIV and the horizontal line. A positive value was defined as the LIV inclining to the convexity of the main curve.
3. UIV translation (Fig. 4a): defined as the distance from the center of UIV to the CSVL. A positive value was defined as the UIV translating to the convexity of the main curve.
4. Instrumentation mass inclination (Fig. 4b): defined by the angle formed between the line drawn from the center of UIV to the center of LIV and the vertical line. A positive value was defined as the instrumentation mass inclining to the convexity of the main curve.

The recruited patients were further assigned into two groups according to whether or not the post-op residual CBD exceeded 3 cm: the balanced group (post-op CBD < 3 cm) and the imbalanced group (post-op CBD ≥ 3 cm).

### Statistical analysis

Data analysis was conducted using statistical software (SPSS 20.0, SPSS Inc., Chicago, IL). Statistical data are presented as the mean ± standard deviation. Independent-sample t test was applied to compare the deformity parameters between the balanced and imbalanced groups. Comparisons between post-op and follow-up parameters were made by paired t test. Based on our clinical experience and meanwhile to avoid the pitfall of multicollinearity between parameters, the pre- to post-operative changes of following parameters including main curve Cobb angle, instrumentation mass inclination, UIV tilt + LIV tilt, UIV translation were selected to explore whether they could contribute to the post-operative correction of CBD using multiple linear regression analysis. A *p* value < 0.05 was considered statistically significant.

## Results

Five patients were found to have the solid tumor around the apical region and the paraspinal nodular neurofibromas was found in all patients. Besides, no patients were detected to have intraspinal neurofibromas from the MRI scan.

### Surgical outcomes

The pre-op, post-op and final Cobb angle of main curve, kyphosis and CBD for thoracic and thoracolumbar/lumbar groups were shown in Table 2. The incidence of CCI immediately after surgery was 0.0 and 63.6% for thoracic and thoracolumbar/lumbar groups, respectively. Both Cobb angle of main curve and regional kyphosis were significantly corrected and well-maintained during follow-up (Table 2). The post-op CBD in thoracolumbar/lumbar group didn’t improve significantly (39.6 ± 12.5 mm vs. 33.6 ± 18.7 mm, *p* = 0.380). However, this post-op CBD got compensated spontaneously during follow-up (33.6 ± 18.7 mm vs. 8.3 ± 11.3 mm, *p* = 0.002).

**Table 2:** Data of deformity correction, dystrophic characteristics and instrumentation constructs for the thoracic and thoracolumbar/lumbar groups

Parameters	Thoracic	Thoracolumbar/lumbar
**Main curve (°)**		
Pre	86.2 ± 23.9	60.6 ± 20.4
Post	44.9 ± 13.9	23.7 ± 14.0
Latest FU	42.9 ± 9.7	24.0 ± 14.3
**Kyphosis (°)**		
Pre	77.5 ± 15.5	37.6 ± 13.2
Post	31.1 ± 4.1	−0.7 ± 16.8
Latest FU	29.4 ± 5.0	−0.5 ± 17.5
**CBD (mm)**		
Pre	54.4 ± 12.8	39.6 ± 12.5
Post	5.4 ± 11.6	33.6 ± 18.7
Latest FU	1.3 ± 5.8	8.3 ± 11.3
**Postoperative CCI (%)**	0	63.6
**Number of dystrophic segments**	5.3 ± 1.0	4.6 ± 1.0
**VRS**	3 (75%)	9 (81.8%)
**Fusion segments**	11.8 ± 1.0	8.5 ± 1.5
**Implant density (%)**	67.7 ± 3.1	78.0 ± 13.2
**Laminar hook ratio (%)**	11.2 ± 11.5	9.7 ± 16.4

*CBD* coronal balance distance, *SVA* sagittal vertical axis, *CCI* convex coronal imbalance, *VRS* vertebral rotatory subluxation

### Comparison between the balanced and imbalanced groups

The imbalanced group was assigned with 7 patients in total (46.7%), all of which were from thoracolumbar/lumbar group. The total number of vertebrae distal to apex was larger in the balanced group (5.6 ± 1.9 vs. 3.3 ± 0.5, *p* = 0.010). The correction rate of CBD was significantly larger in the balanced group (78.4 ± 25.1% vs. -22.9 ± 29.7%, *p* < 0.001). Further comparison revealed a higher correction of main Cobb angle (65.9 ± 9.1% vs. 51.5 ± 37.3%, *p* = 0.040), more tilted instrumentation (10.3 ± 3.6° vs. 3.2 ± 3.1°, *p* = 0.001), reverse tilt and translation of UIV (8.0 ± 2.3° vs. -3.4 ± 5.9°, *p* < 0.001; 35.4 ± 6.9 mm vs. 12.3 ± 13.1 mm, *p* = 0.001) to the convex side in the imbalanced group. These results were in line with the findings that the sum of post-op UIV tilt + LIV tilt was significantly smaller in the balanced group (1.3 ± 9.6° vs. 16.3 ± 7.5°, *p* = 0.005), neutralizing the coronal imbalance (Table 3). Multiple linear regression analysis revealed that △UIV translation was significantly correlated with the correction of CBD (β = 0.832; *p* = 0.030).

**Table 3:** Comparative analysis between the balanced and imbalanced groups

	Balanced group (***n*** = 8)	Imbalanced group (***n*** = 7)	***p***
**Main Curve (°)**			
Preoperative	83.4 ± 18.7	49.1 ± 17.0	**0.001**
Postoperative	40.2 ± 14.8	17.0 ± 7.2	**0.002**
Correction rate (%)	51.0 ± 14.9	65.9 ± 9.1	**0.040**
**CBD (mm)**			
Preoperative	49.0 ± 13.0	37.3 ± 12.9	0.105
Postoperative	9.9 ± 10.5	44.6 ± 12.9	**< 0.001**
Correction rate (%)	78.4 ± 25.1	−22.9 ± 29.7	**< 0.001**
**Instrumentation mass inclination (°)**			
Preoperative	14.7 ± 4.7	8.5 ± 3.4	**0.013**
Postoperative	3.2 ± 3.1	10.3 ± 3.6	**0.001**
**LIV tilt (°)**			
Preoperative	21.0 ± 13.0	19.2 ± 7.6	0.754
Postoperative	4.6 ± 6.6	8.3 ± 7.0	0.316
**UIV tilt (°)**			
Preoperative	−9.5 ± 7.7	−0.7 ± 8.1	**0.049**
Postoperative	−3.4 ± 5.9	8.0 ± 2.3	**< 0.001**
**UIV translation (mm)**			
Preoperative	48.0 ± 14.3	34.7 ± 6.9	**0.045**
Postoperative	12.3 ± 13.1	35.4 ± 6.9	**0.001**
**UIV tilt + LIV tilt (°)**			
Preoperative	11.5 ± 13.0	18.6 ± 9.9	0.257
Postoperative	1.3 ± 9.6	16.3 ± 7.5	**0.005**

*UAI* Upper arc inclination, *LAI* Lower arc inclination, *UAT* Upper arc translation, *LAT* Lower arc translation, *LIV* Lower instrumented vertebra, *UIV* Upper instrumented vertebra

### Follow-up of the imbalanced group

Data of follow-up of the imbalanced group revealed spontaneous compensation of CCI (44.6 ± 12.9 mm vs. 12.6 ± 9.6 mm, *p* = 0.002) (Ratio of CBD < 3 cm: 100%) (Table 4). Decreased tilting of the instrumentation mass and LIV to the convex side (distal compensation) (*p* < 0.05) and increased UIV disc angle (proximal compensation) (*p* = 0.095) accounted for such coronal rebalance. △UIV translation / △CBD (%) during follow-up averaged 63.3 ± 36.2%, which revealed that distal compensation was the mainstay compensative mechanism.

**Table 4:** Follow-up data of the imbalanced group

	Postoperative	Latest FU	*p
**Main curve (°)**	17.0 ± 7.2	16.9 ± 8.3	0.894
**Kyphosis (°)**	0.0 ± 10.7	0.3 ± 12.6	0.975
**Coronal balance (mm)**	44.6 ± 12.9	12.6 ± 9.6	0.002
**Instrumentation mass inclination (°)**	10.3 ± 3.6	4.7 ± 3.4	< 0.001
**UIV translation**	35.4 ± 6.9	16.3 ± 8.5	0.002
**UIV disc angle**	1.6 ± 1.5	5.1 ± 5.1	0.095
**LIV tilt**	8.3 ± 7.0	1.0 ± 7.6	0.008
**Ratio of CBD < 3 cm**	–	100%	–
**△UIV translation / △CBD (%)**	–	63.3 ± 36.2	–

*LIV* Lower instrumented vertebra, *UIV* Upper instrumented vertebra, *CBD* coronal balance distance

## Discussion

CCI associated with DS-NF1 is a unique and rare subtype, and should be distinguished because of the prevailing dystrophic bone phenotype [16, 18, 19]. Strong distal screw purchases are the premise of coronal rebalance yet may not be obtainable for such patients. In this scenario, the management of CCI in DS-NF1 is challenging, especially for those with lower lumbar curve apex. Pelvic fixation can reliably improve the distal screw power, which was a key requirement for several innovative coronal realignment techniques [9, 11, 20, 21]. However, pelvic fixation is not popular for this young patient population. In order to resolve this dilemma, a relatively clear guideline is warranted.

**Fig. 5:**
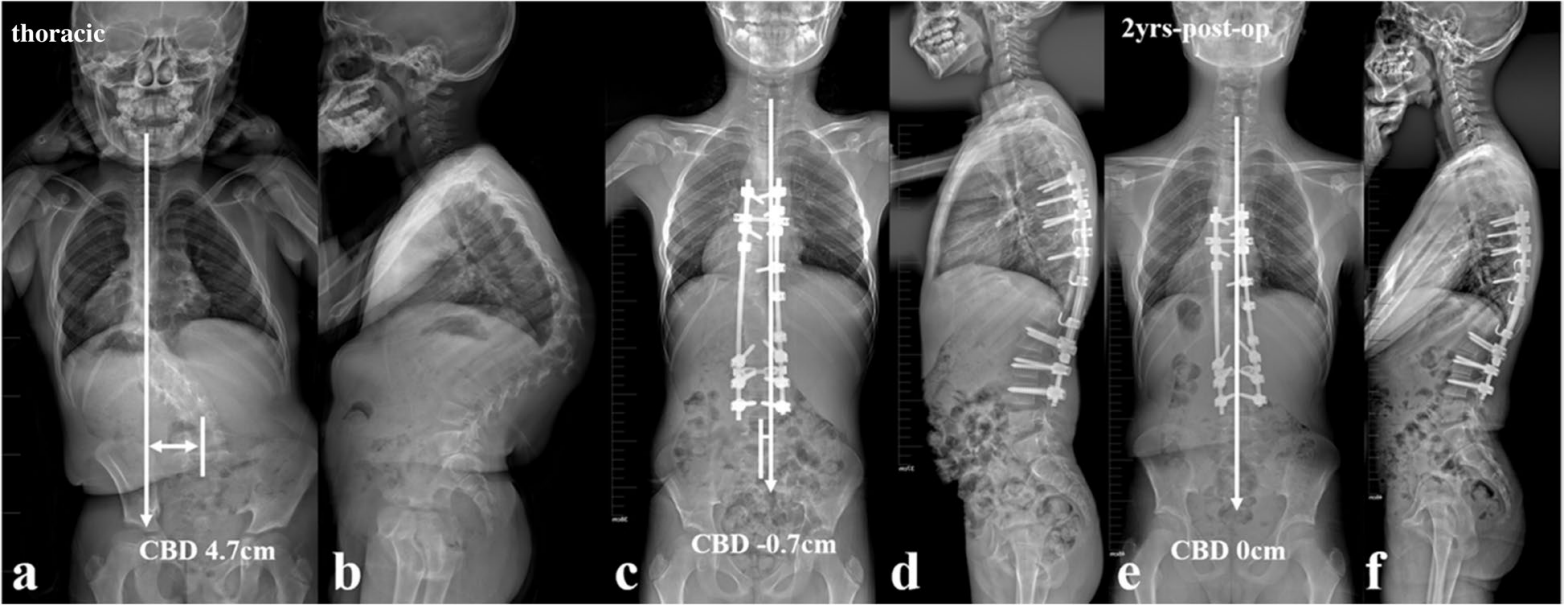
a 12-year-old boy with NF1 related thoracic kyphoscoliosis associated with CCI (thoracic type) (**a**, **b**). Halo gravity traction on wheelchair was prescribed for 1 month. Afterwards, posterior spinal correction and fusion surgery was performed (**c**, **d**), and the CCI improved from 4.7 cm to − 0.7 cm postoperatively (**c**, **d**). At 2-year-follow up, the CBD got further improvement to 0 cm (**e**, **f**)

**Fig. 6:**
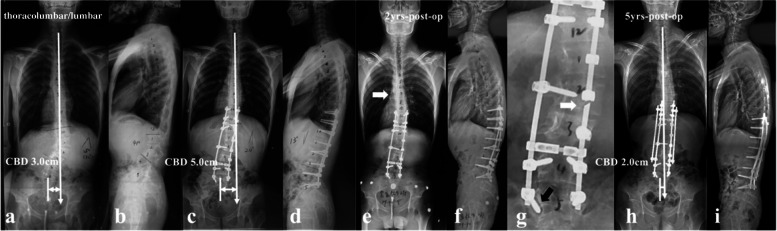
a 26-year-old male patient with NF1 related lumbar kyphoscoliosis associated with CCI (lumbar type) (**a**, **b**). Posterior spinal correction and fusion surgery was performed with over correction of upper hemi-curve, resulting in iatrogenic aggravation of CCI (**c**). Despite spontaneous improvement of CCI during follow-up (**e**), rod fracture occurred at 2-year-follow up (**e**, **f**, **g**, arrow). Revision surgery was performed with satellite rods (four rod constructs) and generous posterior fusion, and no additional rod fracture occurred by 5-year-follow up (**h**, **i**)

The present study represented a homogeneous case series of dystrophic NF1 patients with convex trunk shift, and the incidence reached 8.4%. The stratification of distinguishing thoracic from thoracolumbar/lumbar CCI was important, as the incidence of immediate post-op CCI (≥3 cm) was 0.0 and 63.6% for thoracic and thoracolumbar/lumbar groups, respectively. This separation was essential because of its quite different prognosis. The likely mechanism was that the thoracic CCI was usually associated with sufficient distal non-dystrophic pedicles and reliable distal screw purchases to achieve sufficient correction of distal fractional curve and subsequently a horizontal takeoff (Fig. 5). For thoracolumbar/lumbar CCI, limited and unreliable distal screw purchases were inclined to achieve poor correction of lumbosacral fractional curve and subsequently leave residual takeoff angle, increasing the risk of failure of coronal rebalance (Fig. 6).

The quantitative comparative analysis further revealed that in the imbalanced group, a higher correction of main curve Cobb angle is common, resulting in more tilted instrumentation mass, and revers tilt and translation of UIV to the convex side. This is particularly true for thoracolumbar/lumbar CCI with limited distal screw purchases, more residual lumbosacral takeoff angle and easy over correction of upper hemi main curve. Multiple linear regression analysis further revealed that △UIV translation was a key determinant for correction of CCI.

**Fig. 7:**
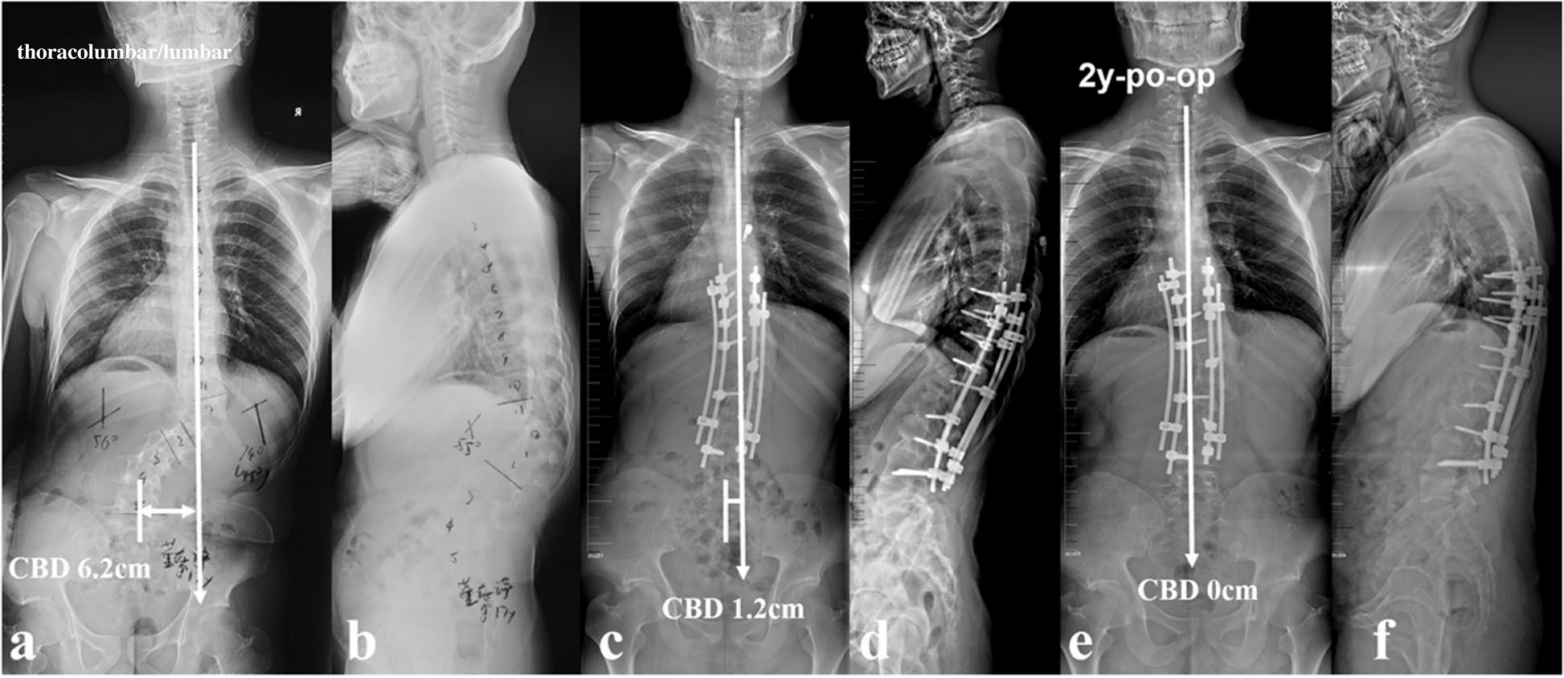
a 17-year-old boy with NF1 related thoracolumbar kyphoscoliosis associated with CCI and vertical straight morphology of upper hemi curve (thoracolumbar type) (**a**, **b**). Over correction maneuvers were avoided for main curve. Fine-tuning using concave compression/convex distraction and coronal rod bending in upper hemi-curve was performed to increase the coronal compensation, resulting in opposite tilt of UIV and LIV with similar magnitude (**c**, **d**). At 2-year-follow up, the CBD got further improvement to 0 cm (**e**, **f**)

Based on the lessons learned from this rare case series, tips for implementing better correction maneuvers when treating this particular patient group were summarized. Rod insertion with derotation and compression maneuvers firstly on the convex side was well known to be beneficial for correction of main curve. However, over correction was easy to occur with simultaneous reverse tilt and translation of UIV to the convex side, resulting in inadvertent aggravation of CCI, particularly for those with thoracolumbar/lumbar apex and vertical proximal hemi-curve. Contrarily, if the rod was firstly inserted on the concave side using translation rather than derotation and cantilever as main correction technique, the risk of over correction of main curve was relatively small, reducing the risk of CCI aggravation. The position of UIV, being represented by its tilt and translation, was of valuable information in evaluating whether or not the coronal rebalance failed intraoperatively. If the UIV tilt and translate to convex side, fine-tuning using concave compression/convex distraction and coronal rod bending in upper hemi-curve region were beneficial to increase the coronal compensation (Fig. 7). Finally, a vertical morphology of instrumentation being confirmed by intraoperative fluoroscopy was essential for reliable coronal realignment.

**Fig. 8:**
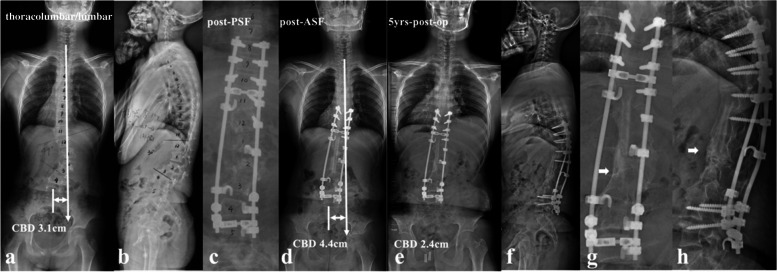
an 11-year-old boy with NF1 related lumbar kyphoscoliosis associated with CCI (lumbar type) (**a**, **b**); Stage 1 posterior spinal correction and fusion surgery using hybrid implants (screws and hooks) was performed with low screw density at apical region (**c**), followed by stage 2 supplementary anterior intervertebral fusion utilizing structural fibular allograft (**d**). Over correction of upper hemi curve resulted in aggravation of CCI (4.4 cm). The patient experienced spontaneous improvement of CBD at 5 years follow-up (**e**). Despite low implant density, no implant failure occurred because the fusion of the anterior column was good (**f**, **g**, **h**)

The follow-up data revealed that patients in the imbalanced group experienced spontaneous improvement of CCI over time (Ratio of CBD < 3 cm: 100%). This usually resulted in an acceptable but not satisfying coronal alignment because residual tilting of the instrumentation mass and/or junctional angulation centering around LIV existed (Fig. 8), and might result in implant failure (Fig. 5). Both distal compensation (decreased tilting of the instrumentation mass and LIV to the convex side) and proximal compensation (increased UIV disc angle) accounted for such coronal rebalance, and our data was suggestive that the distal compensation was the mainstay compensative mechanism. This was in line with Bao’s previous finding that LIV at L4 or higher was correlated to a higher chance of spontaneous coronal rebalance [7].

The limitation for this study lied in that the sagittal alignment was not well considered, which might also influence the design of surgical strategy for coronal realignment. In the future, more patients would be included for further comprehensive analysis to amend this issue.

## Conclusion

The choice of coronal rebalance should be directed by the individual situation as the difficulty of coronal rebalance increased with descending apex location. This study presented a thought-provoking case series that illustrated the substantial utility of CCI stratification by apex location as an important preliminary indicator of the inherent difficulty for CCI correction in DS-NF1. Thoracolumbar/lumbar CCI in dystrophic scoliosis was prone to suffer high risk of persistent post-op CCI when pelvic fixation was not planned. Satisfying coronal rebalance should avoid increased UIV tilt and translation to the convex side, tilted morphology of instrumentation and over correction maneuvers for main curve, especially when the morphology of upper hemi-curve was straight and vertical showing no coronal compensation. It was impressive that the CCI had some relief during follow-up, beneficiating from both proximal and distal compensation. Sacrum or pelvic fixation should be taken as a salvage technique for potential instrumentation failure during follow-up.

## Data Availability

The datasets generated and/or analyzed during the current study are not publicly available but are available from the corresponding author on reasonable request.

## Abbreviations

CI: Coronal imbalance
DS-NF1: Dystrophic scoliosis secondary to Type I neurofibromatosis
CCI: Convex coronal imbalance
CBD: Coronal balance distance
CSVL: Center sacral vertical line
